# “Capacity building to implement state of the art surveillance systems for antibiotic consumption and resistance in kosovo”: results of European Union research project in Kosovo

**DOI:** 10.1186/2047-2994-4-S1-P178

**Published:** 2015-06-16

**Authors:** L Raka, H Goosens, G Mulliqi, A Versporten, S Krasniqi, T Kostyanev, A Kurti, C Lammens, A Kurti, F Paasch, V Uka, A Jakupi, A Loku, D Raka

**Affiliations:** 1 National Institute of Public Health of Kosovo, Albania; 2 University of Antwerp, Belgium; 3 National Institute of Public Health of Kosovo, Prishtina; 4 University of Prishtina; 5 Kosovo Agency for Medical Products, Albania

## Introduction

The project “Capacity building to implement state of the art surveillance systems for antibiotic consumption and resistance in Kosovo” was financed by the European Union and implemented by the National Institute of Public Health of Kosovo in collaboration with the University of Antwerp during 2012-2014.

## Objectives

The main objectives of this project were to monitor volumes and patterns of antibiotic use and to establish a comprehensive surveillance system of antibiotic resistant bacteria.

## Methods

Total antimicrobial use data were analysed according to the WHO ATC/DDD methodology and expressed in DDD/1000inhabitants/day (DID). Data on antimicrobial use in hospitals were collected in all seven hospitals of Kosovo using the methodology of a point prevalence survey based on ESAC. Survey of consumption in primary care was retrospective and involved 12 Family Medicine Centres covering all 6 regions of Kosova.

## Results

Total antibacterial use in Kosovo was 26.3 DID. The top antibacterial subgroups were penicillins (12.8 DID, 48.7% of all antibacterials) and other beta-lactam antibacterials (4.9 DID, 18.7%). Kosovo had the highest proportional total parenteral use of cefriaxone in Europe (53.9) and lowest use of systemic antimycotics and antifungals (0.08 DID). Of total number of 1579 enrolled patients in Kosovo hospitals, 769 (48.7%) of them received antibiotics. The most prescribed antibiotic was ceftriaxone (41.5%), followed by gentamycin (12.1%). Antibiotics were administered mainly through parenteral route (73.3%). Empiric antibiotic prescription was higher than etiological (88.28% vs. 11.72%). Of all patients who attended primary care level, 33% received antibiotics. Administration through the parenteral route was 43%. Antibiotic prescription with generic names was noticed only in 31% of cases. The most prescribed antibiotic in primary care level was also ceftriaxone.

## Conclusion

Gathered data will serve as a tool for quality improvement in Kosovo and will support the preparation of guidelines and protocols for prudent use of antibiotics.

## Disclosure of interest

None declared.

